# Supplementary material for: Olfactory receptor OR2AT4 regulates human hair growth
Source: Nat Commun. 2018 Sep 18;9:3624. doi: 10.1038/s41467-018-05973-0 (PMC6143528; doi:10.1038/s41467-018-05973-0)
Supplement: Supplementary file 3 — Supplementary Information [file 41467_2018_5973_MOESM3_ESM.pdf]

Olfactory receptor OR2AT4 regulates human hair growth

Chéret et al.

Supplementary Information

Supplementary Figures

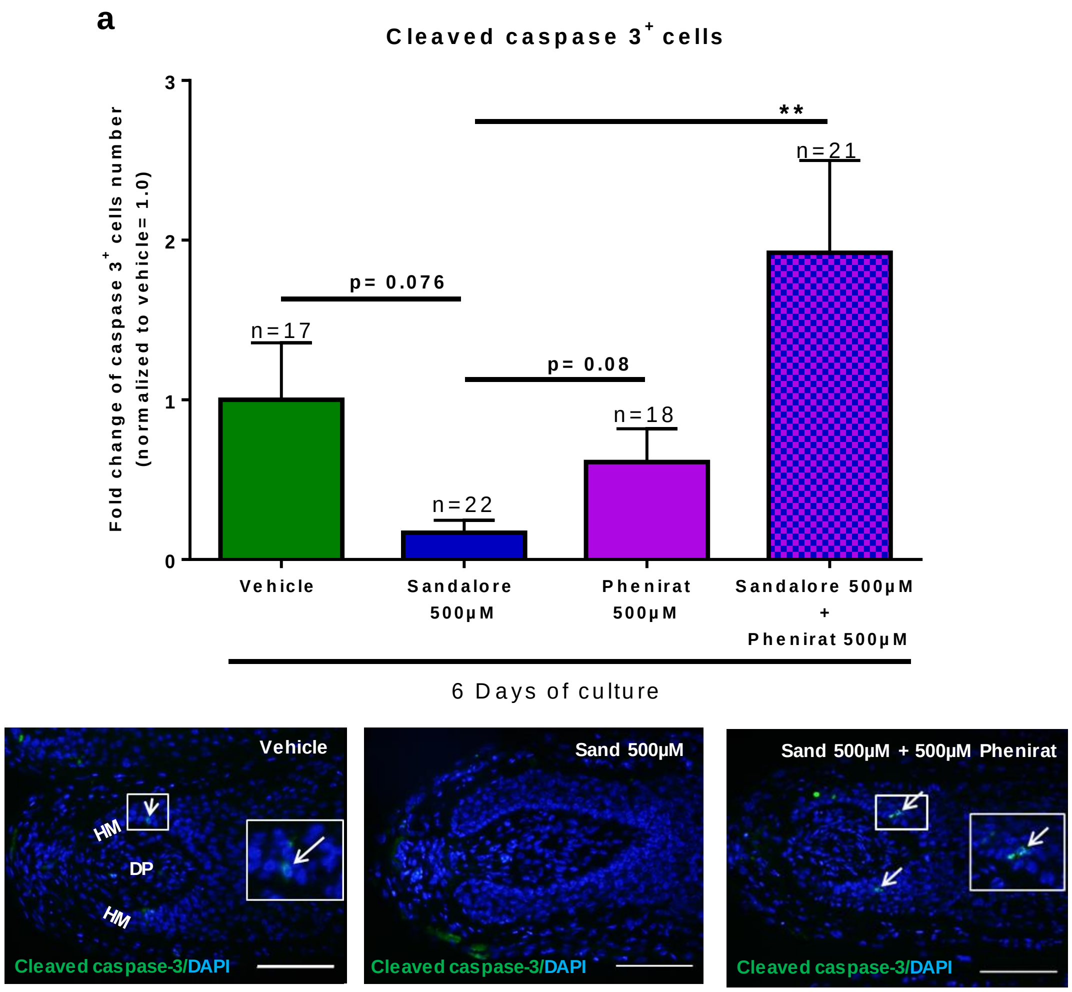

Supplementary Figure 1: Sandalore® slightly decreases the number of apoptotic hair matrix keratinocytes

The number of cleaved caspase-3+ cells (white arrows) in the hair matrix was evaluated in the hair bulb of all treated and vehicle HFs (a). Representative pictures of cleaved caspase-3 staining. Mean±SEM, n=17-22 HFs from 3 donors (independent experiments), Kruskal-Wallis test (P=0.0253) and Dunn's multiple comparisons test as post hoc test, **P<0.01. DP, dermal papilla; HM, hair matrix. Scale bar: 100µm.

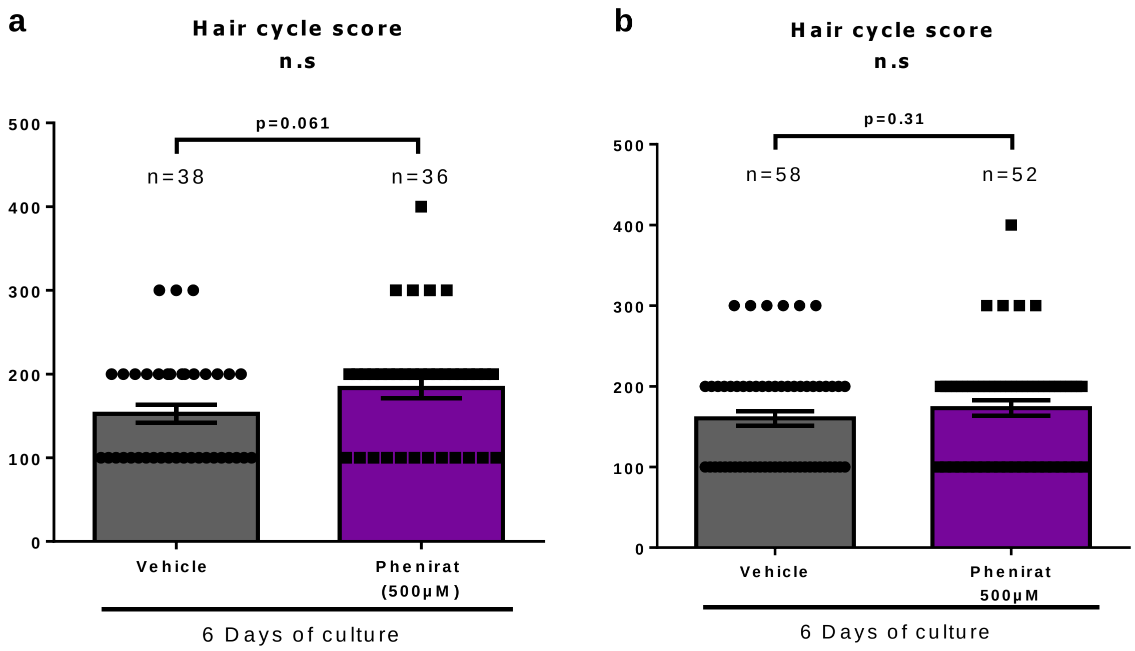

Supplementary Figure 2: Phenirat® treatment alone tendentially induces premature catagen development in HF

It is conceivable that the small difference between vehicle and Phenirat® alone may be due to the fact that the level of (unknown) endogenous OR2AT4 ligands still present in organ-cultured control HFs did not suffice to permit Phenirat® to efficiently compete with endogenous ligands at the receptor in our model; moreover, currently available literature data do not permit one to formally exclude that Phenirat® might also bind to other ORs, thus potentially counteracting hair growth-inhibitory effects it exerts via binding to OR2AT4.

Hair cycle score was evaluated in treated and vehicle HFs after 6 days of culture using Ki-67/TUNEL immunofluorescence and Masson Fontana histochemistry (1). Mean±SEM, n=36-38 HFs from 3 donors (independent experiments), unpaired Student's t-test, ns: not significant (a), Mean±SEM, n=52-58 HFs from 7 donors (independent experiments), Mann-Whitney test, ns: not significant (b).

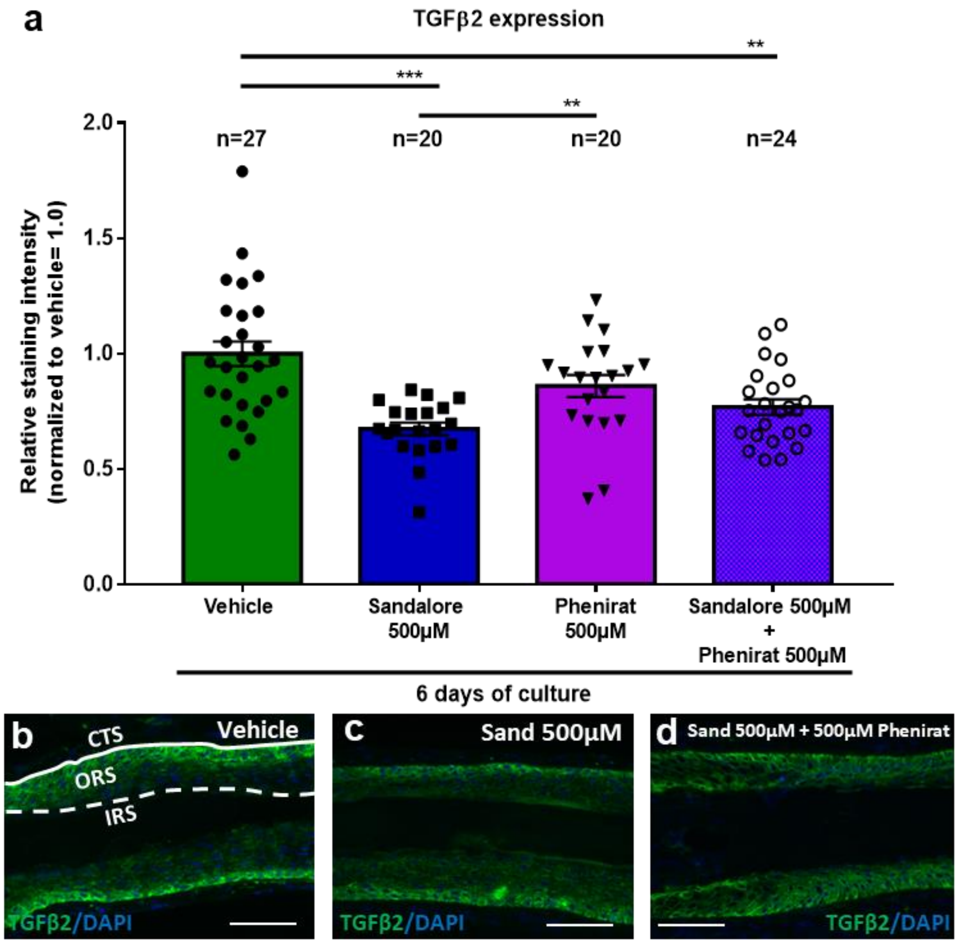

Supplementary Figure 3: Sandalore® significantly decreases TGFβ2 expression in the ORS keratinocytes and can be reversed by the co-administration of Sandalore® with Phenirat®

TGFβ2 expression was measured in ORS keratinocytes in treated and vehicle HFs (a). Representative pictures of TGFβ2 immunofluorescence. TGFβ2 expression was measured using Image J. Mean±SEM, n=20-27 HFs pooled from 3 donors (independent experiments), Kruskal-Wallis ($P=0.004$) and Dunn's multiple comparisons test as post hoc test, *$P<0.05$, ***$P<0.001$. CTS, connective tissue sheath; IRS, inner root sheath; ORS, outer root sheath. Scale bar: 100µm.

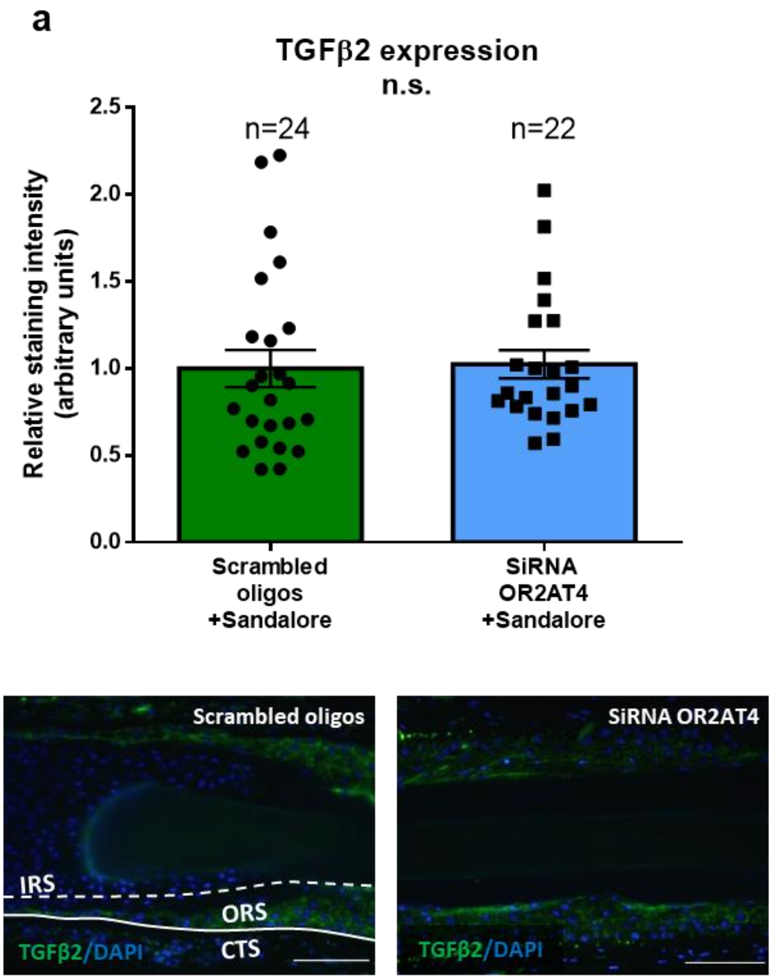

Supplementary Figure 4: OR2AT4-knockdown does not affect intrafollicular TGFβ2 protein expression under Sandalore® stimulation

TGFβ2 protein expression was measured by quantitative immunohistomorphometry (image J) in the proximal ORS of HFs treated with OR2AT4-siRNA or scrambled oligos (a). Representative images of TGFβ2 immunofluorescence in the ORS of HFs. Mean±SEM, n=22-24 HFs from 3 donors (independent experiments), Mann-Whitney test, n.s.: not significant. CTS, connective tissue sheath; IRS, inner root sheath; ORS, outer root sheath. Scale bar: 100µm.

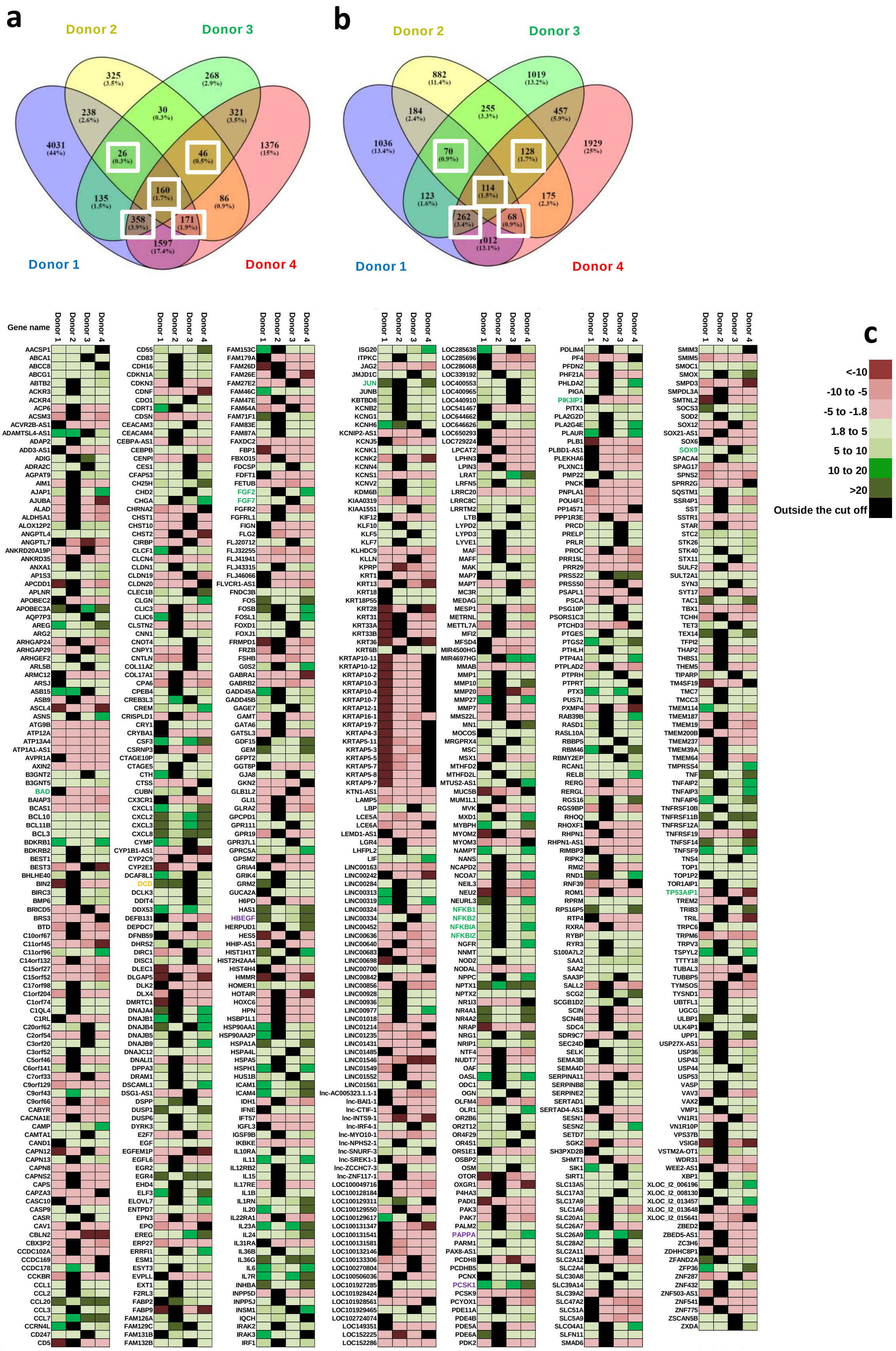

Supplementary Figure 5: Microarray-based analysis of HFs after 6 hours stimulation with Sandalore® (500µM).

Venny diagrams (2) showed the up- (a) and down-regulated (b) genes (Cut off: fold change >-1.8 or >+1.8 & equidirectional changes). White squares indicated genes up- and down-regulated in at least 3

of 4 donors (independent experiments). The heatmap showed the list and the expression level of the most up and down-regulated genes (Cut off: fold change >-1.8 or >+1.8 & equidirectional changes) in at least 3 of 4 donors (independent experiments) (c). The gene in bold are related to the different pathways involved after OR2AT4 activation (Green: apoptosis-related; orange: dermcidin-related; and violet: IGF-related).

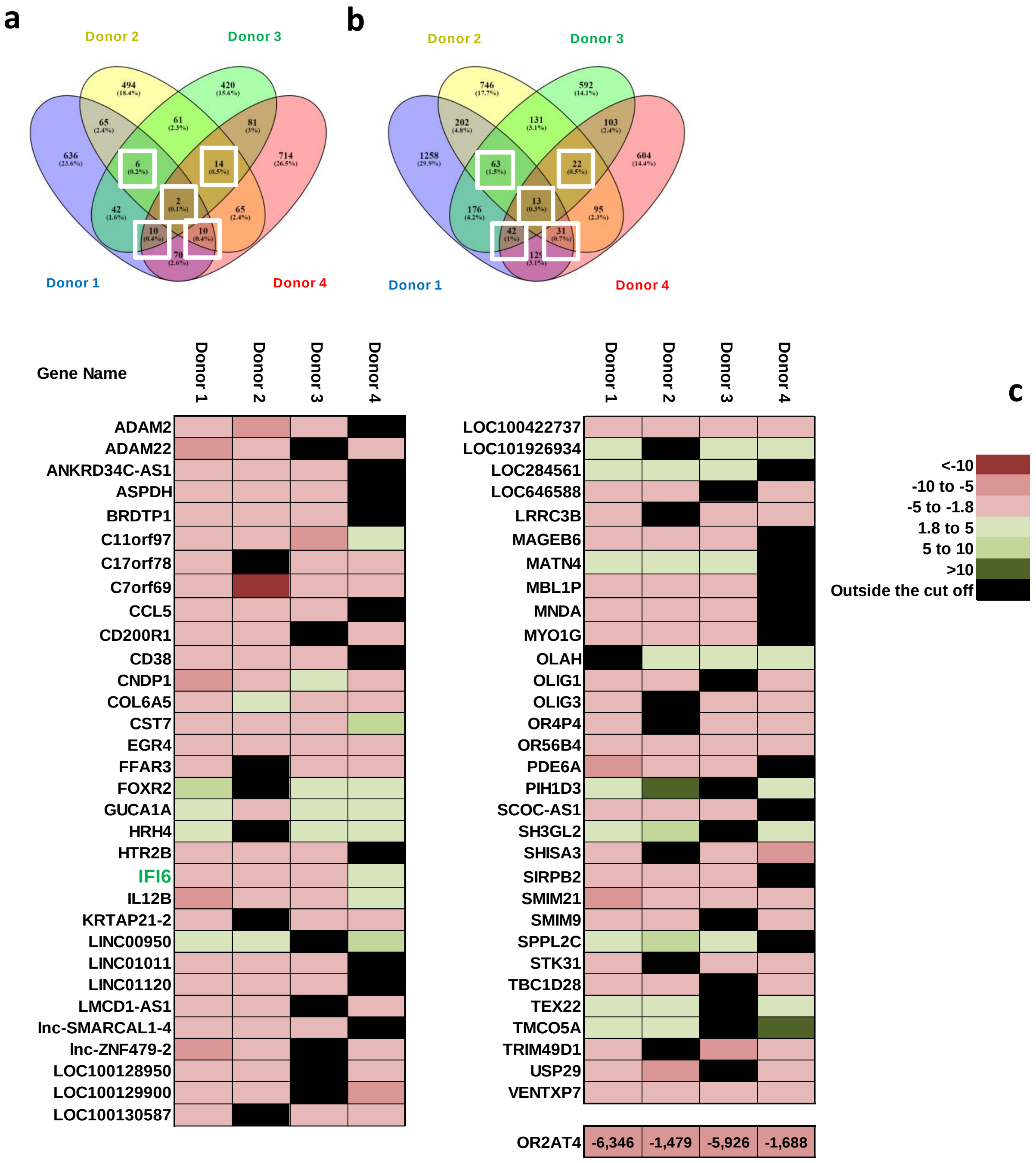

Supplementary Figure 6: Microarray-based analysis of HFs after OR2AT4 knock-down.

Venny diagrams (2) showed the most up- (a) and down-regulated (b) genes (Cut off: fold change >-1.8 or >+1.8 & equidirectional changes). White squares indicated genes up- and down-regulated in at least 3 of 4 donors (independent experiments). The heatmap showed the list and the expression level of the most up and down-regulated genes (Cut off: fold change >-1.8 or >+1.8 & equidirectional changes) in at least 3 of 4 donors (independent experiments) (c). The gene in bold green is related to the apoptosis pathway involved after OR2AT4 activation.

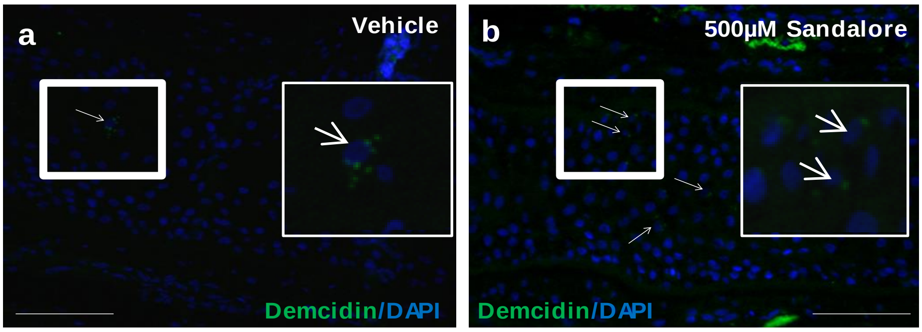

Supplementary Figure 7: Sandalore® increase dermcidin protein expression in the epithelium of human scalp HF

Dermcidin (white arrows) protein is indeed up-regulated by Sandalore® (e.g. 1 positive cell for vehicle vs >4 positive cells for Sandalore®-treated HFs in the representative pictures presented here) in the epithelium of human scalp hair follicles (a-b). Representative images of dermcidin immunofluorescence in the epithelium of vehicle (a) and Sandalore®-treated (b) HFs from 3 different donors (N=3 independent experiments). Scale bar: 100μm.

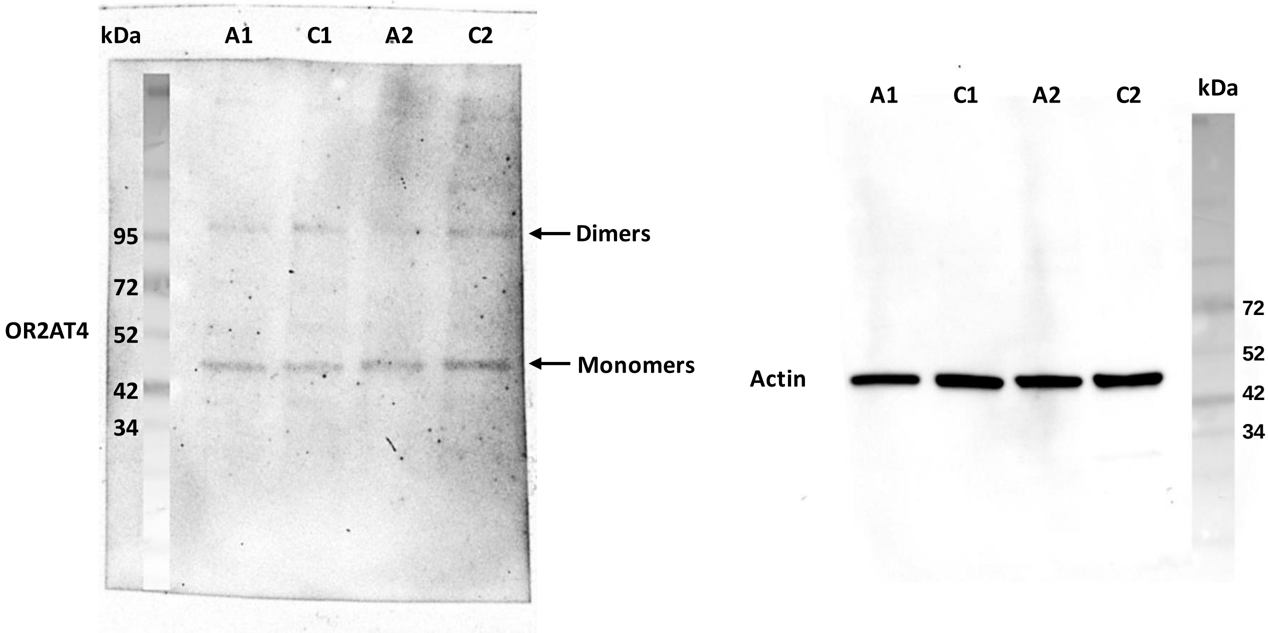

Supplementary Figure 8: (uncropped WB images related to Figure 2f)

Supplementary Notes

Supplementary Note 1:

Given the extremely scarce availability of human scalp HFs, classical dose-response studies are generally not possible, and the test agent concentrations used must be carefully selected. The choice of 500µM was based on the previously established dose–response curve of Sandalore®-induced Ca2+ signals on HaCaT cells and human keratinocytes (3) where the IC50 was 430µM. In this previous study, we had also shown that, at 500µM, the proliferation and migration of human epidermal keratinocytes is significantly increased compared to vehicle and that the reepithelialisation of experimental human skin wounds *ex vivo* is significantly accelerated, while concentrations below 100µM failed to activate OR2AT4 (3). In additional preparatory experiments, we had also compared the effects of 50 and 500µM of Sandalore® on hair matrix keratinocyte proliferation/apoptosis as well as on OR2AT4 expression; again, this demonstrated maximal effects at 500µM (Fig. 3a-e). In addition, toxicological screens such as genotoxicity and cytotoxicity test *in vitro*, and carcinogenicity test *in vivo*, Sandalore® did not exhibit carcinogenicity (4). Furthermore, Sandalore® is not listed as carcinogenic substance by the OSHA (Occupational Safety and Health Administration (29 CFR 1910.1001-1050)).

Supplementary Note 2:

Phenirat® is known to be a competitive antagonist of OR2AT4 only in the presence of Sandalore® (3). Although the results deriving from the pool data of the three independent experiments (3 donors) presented during the first submission do not support the hypothesis that Phenirat® itself may promote premature catagen development (see Fig. 1b), we have clarified this in four additional *ex vivo* experiments. This showed that Phenirat® alone tendentially promotes premature catagen development ex vivo, even though this did not reach significance due to the high degree of interindividual variability in human HF responses to Phenirat® seen in these experiments (see Supplementary Fig. 2a). This new data together with our old data (see supplementary Fig. 2b) suggest that, overall, Phenirat® operates only as a weak inhibitor of human hair growth *ex vivo* in the absence of Sandalore®. It is conceivable that the small difference between vehicle and Phenirat® alone may be due to the fact that high levels of (unknown) endogenous OR2AT4 ligands still present in organ-cultured control HFs did not suffice to permit Phenirat® to efficiently compete with endogenous ligands at the receptor in our model; moreover, currently available literature data do not permit one to formally exclude that Phenirat® might also bind to other ORs, which might counteract the hair growth-inhibitory effects it exerts via binding to OR2AT4.

Supplementary Note 3:

The concentration of IGF-1 neutralizing antibody used in our study has been shown to neutralize 5.0 ng/ml of IGF-1 (see datasheet of the IGF-1 neutralizing antibody, Abcam, ab9572). We have purposely used this concentration of the neutralizing antibody to neutralize only a certain percentage of the endogenous IGF-1 present in the hair follicle *ex vivo*, to be able to have at least some hair follicles remaining in anagen after the treatment (IGF-1 is required for maintaining human HFs in anagen [5]).

As one can appreciate in Fig. 1e, the selected concentration of IGF-1 neutralizing antibody was sufficient to promote premature catagen induction by reducing the concentration of available endogenous IGF-1 for the binding to the receptor (see hair cycle staging graph). Instead, Sandalore®, which increases endogenous IGF-1 protein expression and prolongs anagen (Fig. 1b, d), significantly counteracts premature catagen induction by the selected concentration of IGF-1 neutralizing antibody. Therefore, this data strongly suggests that the anagen-prolonging effect of OR2AT4 activation by Sandalore® is mainly, but possibly not exclusively, due to the increased IGF-1 secretion.

Supplementary Discussions

Supplementary Discussion 1:

Mice have an OR2AT4 homolog which might exert a conserved role in HF growth. In fact, OR2AT4 is one of the most homologous ORs between human and mice (88% conserved), with the highest known OR homology between mice and humans being about 94% (source: The Human Olfactory Data Explorer). Therefore, it is theoretically conceivable that the murine OR2AT4 receptor might be activated by the same ligands and plays a similar role in murine HF biology as it does in human scalp HFs. However, the molecular receptive field of murine OR2AT4 would first have to be rigorously tested, because the exchange of just one of its about 320 amino acids is sufficient to greatly change the spectrum of OR ligands recognized greatly (6). Therefore, it remains quite uncertain and as yet entirely unproven whether the murine OR2AT4 receptor recognizes the same ligands as its human counterpart.

Supplementary Discussion 2:

Previously, we had addressed the specificity and receptor dependence of Sandalore® effects at the relatively high concentration tested here in human HaCaT cells and primary human epidermal keratinocytes (3). This had demonstrated that after 5 days of Sandalore® stimulation in the presence of specific antagonist of OR2AT4 or of relevant signalling pathway blockers (adenylyl cyclase inhibition by MDL-12.330A or SQ-22536; CNG channel inhibition by L-cis Diltiazem), the Sandalore® effect in HaCaT cells or primary human epidermal keratinocytes was completely blocked, without any effects on cell morphology or any other cell biology parameter evaluated. In addition, co-application of the specific OR2AT4 antagonist Phenirat® with Sandalore® (1:1, 500 mM) completely blocked Sandalore®-induced calcium signals in HaCaT cells (3), indicating that the effects of Sandalore® are based on the activation of OR2AT4 in keratinocytes. Most importantly, silencing OR2AT4 in organ-cultured HFs clearly showed that – notably in the presence of high-dose Sandalore® – OR2AT4 knock-down exerted the opposite effects of agonist treatment alone and profoundly inhibited both hair growth (i.e. OR2AT4 siRNA shortened anagen and prematurely induced catagen and up-regulated hair matrix keratinocyte apoptosis) and intrafollicular IGF-1 production (see Fig. 5).

Supplementary References

1: Samuelov, L. *et al.* (2012) P-cadherin regulates human hair growth and cycling via canonical Wnt signaling and transforming growth factor-β2. *J Invest Dermatol.* 132(10):2332-41

2: Chen, H. & Boutros, PC. (2011) VennDiagram: a package for the generation of highly-customizable Venn and Euler diagrams in R. *BMC Bioinformatics.* 12:35.

3: Busse, D. *et al*. (2014) A synthetic sandalwood odorant induces wound-healing processes in human keratinocytes via the olfactory receptor OR2AT4. *J Invest Dermatol.* 134(11):2823-32.

4: Burdock, G. A. & Carabin, I. G. (2008) Safety assessment of sandalwood oil (Santalum album L.). *Food Chem Toxicol.* 46(2):421-32.

5: Philpott, M. P. Sanders, D. A. & Kealey, T. (1994) Effects of insulin and insulin-like growth factors on cultured human hair follicles: IGF-I at physiologic concentrations is an important regulator of hair follicle growth in vitro. *J Invest Dermatol.* 102(6):857-61.

6: Wolf, S. et al. (2017) Dynamical binding modes determine agonistic and antagonistic ligand effects in the prostate-specific G-protein coupled receptor (PSGR). *Sci Rep.* 7(1):16007.
