# Supplementary material for: Olfactory receptor OR2AT4 regulates human hair growth
Source: Nat Commun. 2018 Sep 18;9:3624. doi: 10.1038/s41467-018-05973-0 (PMC6143528; doi:10.1038/s41467-018-05973-0)
Supplement: Supplementary file 4 — Description of Additional Supplementary Files [file 41467_2018_5973_MOESM4_ESM.pdf]

Description of Additional Supplementary Files

File Name: Supplementary Data 1

Description: Top up and down-regulated genes from microarray data of HFs treated with Sandalore® (500µM) or after OR2AT4 knock-down under Sandalore® stimulation (500µM). Microarray analysis (Agilent system, see supplementary material and methods) was performed on microdissected HFs from 4 different donors (independent experiments). The tables showed the top up and down-regulated genes (Cut off: fold change <-5 or >+5 & equidirectional changes) for organ-cultured human anagen VI scalp HFs after stimulation with the selective OR2AT4 agonist, Sandalore® (500µM) (a) or after OR2AT4 knock-down and stimulation with Sandalore® (b). The genes in bold are related to the different pathways involved after OR2AT4 activation (Green: apoptosis-related; orange: dermcidin-related; and violet: IGF-related).
